# Evaluation of ten (10) SARS-CoV-2 rapid serological tests in comparison with WANTAI SARS-CoV-2 ab ELISA in Burkina Faso, West Africa

**DOI:** 10.1186/s12985-023-02011-4

**Published:** 2023-03-30

**Authors:** Henri Gautier Ouedraogo, Abdou Azaque Zoure, Tegwinde Rebeca Compaoré, Herve Ky, Sylvie Zida, Dezemon Zingué, Oumarou Ouedraogo, Serge Théophile Soubeiga, Tani Sagna, Charlemagne Dabiré, Dinanibè Kambiré, Dramane Zongo, Albert Théophane Yonli, Abdoul Rahamani Nikiema, Désiré Nezien, Gnintassa Cyrille Bansé, Brice Wilfried Bicaba, Sophie Perier, Charles Sawadogo, Zakariya Yabre, Lassana Sangare

**Affiliations:** 1 Biomedical Research Laboratory (LaReBio)/Biomedical and Public Health Department, Institut de Recherche en Sciences de la Santé (IRSS), Centre National de la Recherche Scientifique et Technologique (CNRST), 03 BP 7047 Ouagadougou, Burkina Faso; 2 Unité de Formation et de Recherche en Sciences de la Santé (UFR-SDS), Université Joseph Ki-Zerbo, Ouagadougou, Burkina Faso; 3 Direction des Laboratoires de Biologie Médicale (DLBM), Ministère de la santé et de l’Hygiène publique, Ouagadougou, Burkina Faso; 4 Institut National de Santé Publique (INSP), Ministère de la santé et de l’Hygiène publique, Ouagadougou, Burkina Faso; 5 Centre de Recherche Biomoléculaire Pietro Annigoni (CERBA), Ouagadougou, Burkina Faso; 6 Laboratoire National de Santé Publique (LNSP), Ministère de la Santé et de l’Hygiène Publique, Ouagadougou, Burkina Faso; 7 Clinique Notre Dame de la Paix, Ouagadougou, Burkina Faso; 8 Centre des Opérations de Réponses aux Urgences Sanitaires (CORUS) /Ministère de la Santé et de l’Hygiène Publique, , Ouagadougou, Burkina Faso; 9 Clinton Health Access Initiative (CHAI), Regional offfice, Dakar, Senegal

**Keywords:** COVID-19, SARS-CoV-2, Antibody, Rapid serological diagnostic test, Point-of-care, ELISA

## Abstract

**Background:**

The aim of this study was to evaluate the performance of ten (10) SARS-CoV-2 serological rapid diagnostic tests in comparison with the WANTAI SARS-CoV-2 Ab ELISA test in a laboratory setting.

**Materials and methods:**

Ten (10) SARS-CoV-2 serological rapid diagnostic tests (RDTs) for SARS-CoV-2 IgG/IgM were evaluated with two (2) groups of plasma tested positive for one and negative for the other with the WANTAI SARS-CoV-2 Ab ELISA. The diagnostic performance of the SARS-CoV-2 serological RDTs and their agreement with the reference test were calculated with their 95% confidence intervals.

**Results:**

The sensitivity of serological RDTs ranged from 27.39 to 61.67% and the specificity from 93.33 to 100% compared to WANTAI SARS-CoV-2 Ab ELISA test. Of all the tests, two tests (STANDARD Q COVID-19 IgM/IgG Combo SD BIOSENSOR and COVID-19 IgG/IgM Rapid Test (Zhejiang Orient Gene Biotech Co., Ltd)) had a sensitivity greater than 50%. In addition, all ten tests had specificity greater than or equal to 93.33% each. The concordance between RDTs and WANTAI SARS-CoV-2 Ab ELISA test ranged from 0.25 to 0.61.

**Conclusion:**

The SARS-CoV-2 serological RDTs evaluated show low and variable sensitivities compared to the WANTAI SARS-CoV-2 Ab ELISA test, with however a good specificity. These finding may have implications for the interpretation and comparison of COVID-19 seroprevalence studies depending on the type of test used.

## Introduction

The recommended reference technique for the diagnosis of COVID-19 is “Reverse transcription polymerase chain reaction” (RT-PCR) test on respiratory samples [1, 2]. The diagnostic result by this technique is usually obtained within four hours. The high cost and time constraints associated with RT-PCR have led to the emergence of alternative diagnostic methods, including antigenic tests or serological tests for the diagnosis of SARS-CoV-2 [1]. These tests are generally based on the lateral flow immunochromatographic principle, which is simple to use and provides results in less than 30 min, or the automated enzyme-linked immunosorbent technique with a delay of approximately 1.5 min for results [1, 3]. Automated serological tests can be categorized according to the reading platforms used to detect SARS-CoV-2 antibodies [4]. They include Enzyme-Linked Immunosorbent Assay (ELISA) and Sandwich Enzyme Immunoassay with Final Fluorescence Detection (FEIA), as well as Chemiluminescence Immunoassay (CLIA), Chemiluminescent Microparticle Immunoassay (CMIA) and Electrochemiluminescence Immunoassay (ECLIA) [4]. These require specific laboratory equipment that are not available in resource-limited settings, often resulting in the use of rapid antibody diagnostic tests both in the laboratory and in seroprevalence studies (Zhao et al., 2020).

In addition, evaluations’ results in the literature show, however, a great variability in the diagnostic performance of commercially available serological tests [5–11]. The vast majority of the evaluations performed have been carried out by comparison with RT-PCR [8, 9, 12–14]. One of the main limitations of RT-PCR is the risk of false negative and sometimes false positive results. [15, 16]. False-negative or false-positive results of RT-PCR tests may result in a decrease in the specificity and sensitivity, respectively, of the serological tests being evaluated. In addition to the risk of false-positive RT-PCR results [17–19], studies have shown that some patients infected with SARS-CoV-2 do not produce antibodies [20, 21]. The use of RT-PCR as a reference may therefore lead to an underestimation of the sensitivity of serological tests if such patients are included in the sample panel used. The use of a reliable serological test could help to eliminate these undetectable SARS-CoV-2 specific antibody producing patients from the evaluation sample panels. Thus, this study proposed to evaluate the performance of ten (10) immunochromatographic tests for the rapid detection of SARS-CoV-2 antibodies in comparison with the WANTAI SARS-CoV-2 Ab ELISA, one of the tests that has shown good performance through several independent evaluations in the literature [9, 10].

## Materials and methods

### Study design

This was an evaluation of the COVID-19 IgG/IgM rapid serological diagnostic tests at the Biomedical Research Laboratory (LaReBio), one of the COVID-19 diagnostic laboratories in Ouagadougou, Burkina Faso.

### Composition of the sample panel

The rapid serological tests were evaluated using two (2) panels of human plasma previously tested for the presence or absence of SARS-CoV-2 antibodies with the WANTAI SARS-CoV-2 Ab ELISA kit on the “Elisys Uno” automated machine (Human, Germany). All plasma samples were collected between December 2020 and April 2021, before the introduction of vaccination against COVID-19 in Burkina Faso. Venous blood samples were collected using EDTA tubes and centrifuged at 3000 rpm for 10 min to separate the plasma. The plasma was then used to perform the serological tests. Blood samples were collected independently of the history of SARS-CoV-2 infection.

### Panels of positive and negative samples

The positive panel consisted of 157 SARS-CoV-2 antibodies positive plasma with the WANTAI SARS-CoV-2 Ab ELISA. The negative panel consisted of 157 SARS-CoV-2 antibodies negative plasma confirmed by the WANTAI SARS-CoV-2 Ab ELISA test.

### Index tests (serological tests in evaluation)

All SARS-CoV-2 serological rapid diagnostic tests (RDTs) evaluated were rapid lateral flow immunochromatographic tests for the qualitative detection of IgG/IgM antibodies to SARS-CoV-2 in either whole blood and/or plasma and serum [22]. They consist of a test membrane and a plastic cassette. The test cassette displays the letters C (control line), G (the IgG test line) and M (the IgM test line) on the right side of the reading window and the letter S (the sample well) above the sample well of the cassette. To use the test, the sample is applied first to the sample well S, then 2–3 drops of the buffer solution will be added. The sample and buffer mixture migrates along the test membrane to the reading window. On the nitrocellulose membrane inside the reading window, human anti-IgG and anti-IgM antibodies are present in the G-zone and M-zone respectively, and a goat anti-rabbit antibody is present in the C-zone. If the sample is positive for SARS-CoV-2 IgG, the G line will appear. If the sample is positive for SARS-CoV-2 IgM, the M line will appear. The validity of the test is indicated by the appearance of the C line regardless of the G and/or M result [23].

The ten index serological tests were: (T1) COVID-19 IgG/IgM Rapid Test: (Whole blood/Serum/Plasma) Sienna TM; (T2) COVID-19 BSS (IgG/IgM) BIOSYNEX; (T3) COVID-19 IgG/IgM cassette (whole blood/serum/plasma) ACCU-Tell, (T4) COVID-19 IgG/IgM Rapid Test (whole blood/serum/plasma) InnoScreen™; (T5) COVID-19 IgG/IgM Rapid Test Device (WB/S/P) Safecare Bio-Tech; (T6) COVID-19 IgG/IgM Rapid Test (Whole blood/Serum/Plasma); (T7) 2019-nCOV IgG/IgM Rapid test Device Hangzhou Realy Tech; (T8) COVID-19 IgG/IgM Rapid Test Cassette (Whole blood/Serum/Plasma) Zhejiang Orient Gene Biotech Co.,Ltd (T9) Standard Q COVID-19 IgM/IgG Combo SD Biosensor; (T10) Panbio COVID-19 IgG/IgM RAPID test device (fingerstick whole blood/venous whole blood/serum/plasma Abbott; The characteristics of these tests according to their manufacturers are shown in Table 1.

**Table 1:** Characteristics of the reference test (WANTAI SARS-CoV-2 Ab ELISA) and index tests (RDTs) according to the manufacturers

Characteristics	Reference test		Index tests									
	WANTAI SARS-CoV-2 Ab ELISA		COVID-19 IgG/IgM Rapid Test: (Whole blood/Serum/Plasma)	COVID-19 BSS (IgG/IgM)	COVID-19 IgG/IgM CASSETTE (Whole blood/Serum/Plasma)	COVID-19 IgG/IgM Rapid Test (Whole blood/Serum/Plasma)	COVID-19 IgG/IgM RAPID TEST DEVICE (WB/S/P)	COVID-19 IgG/IgM Rapid Test (Whole blood/Serum/Plasma)	2019-nCOV IgG/IgM Rapid test Device	COVID-19 IgG/IgM Rapid Cassette test (Whole blood/Serum/Plasma)	STANDARD Q COVID-19 IgM/IgG Combo	Panbio COVID-19 IgG/IgM RAPID TEST DEVICE
Manufacturer’s name	Beijing Wantai Biological		Sienna TM	Biosynex	ACCU-Tell	InnoScreen™	SafeCare BioTech	Missing	Hangzhou Realy Tech	Zhejiang Orient Gene Biotech Co.,Ltd	SD Biosensor	Abbott
Format	Liquid		Cassette	Cassette	Cassette	Cassette	Cassette	Cassette	Cassette	Cassette	Cassette	Cassette
Principle of the test	Antigen “sandwich” enzyme immunoassay		Immunochro matography	Immunochromatography	Immunochromatography	Immunochromatography	Immunochromatography	Immunochromatography	Immunochromatography	Immunochromatography	Immunochromatography	Immunochromatography
Antibodies detected	Total Ab		IgM and IgG	IgM and IgG	IgM and IgG	IgM and IgG	IgM and IgG	IgM and IgG	IgM and IgG	IgM and IgG	IgM and IgG	IgM and IgG
Product code	WS-1096		10,222 A	---	ABT-IT-B352	WCOV-23 M	--	--	K4602160	GCCOV-402a	Q-NCOV-01 C QC05020028A	ICO-T402P
Lot number	NCOA20210501		20,050,504	COV20040078	2,020,032,705	SR202004013	NC020032603	2,020,025	N01G16T	2,004,155	2020.06.01	COV0042014
Samples used	serum/ plasma		Whole blood/serum/ plasma	Whole blood/serum/ plasma	Whole blood/serum/ plasma	Whole blood/serum/ plasma	Whole blood/serum/ plasma	Whole blood/serum/ plasma	Whole blood/serum/ plasma	Whole blood/serum/ plasma	Whole blood/serum/ plasma	Whole blood/serum/ plasma
Storage (°C)	+ 2–8°		+ 2–30°	+ 2–30°	+ 2–30°	+ 2–30°	+ 2–30°	+ 2–30°	+ 2–30°	+ 2–30°	+ 2–30°	+ 2–30°
Reading/Interpretation	plate reader, wavelength 450/600 ~ 650 nm		Visual	visual	visual	visual	visual	visual	visual	visual	visual	visual
Time to obtain results (min)	90		10	10	10	15	10	10	10	2	10	10
Time to read results (max)	10		20	20	20	20	15	15	15	15	15	20
Composition of the kit	a		c	c	c	c	c	c	c	c	c	c
Consumables required but not supplied	b		b	b	b	b	b	b	b	b	b	b
Sensitivity according to the manufacturer (%)	98.72 (> 15 days from onset of symptoms)		91.76 (IgM) and 88.24 (IgG)	100 (IgM) and 91.8 (IgG)	91.8 (IgM) and 100 (IgG)	93.7 (IgM) and 98.8 (IgG)	Not provided	Not provided	92 (IgM) and 96 (IgG)	87.9 (IgM) and 97.2 (IgG)	94.51 combined sensitivity	56.25 (IgM) and 95.83 (IgG)
Specificity according to the manufacturer (%)	98.60		99.16 (IgM) and 99.46 (IgG)	99.5 (IgM) and 99.2 (IgG)	99.2 (IgM) and 99.5 (IgG)	97.7 (IgM) and 98.7 (IgG)			100 (IgM) and 100 (IgG)	100 (IgM) and 100 (IgG)	95.74 Combined specificity	94 (IgM) and 100 (IgG) Combined specificity

*a*: Microwell plate, Cardboard plate cover, Negative calibrator, Positive calibrator, Horseradish peroxidase-conjugated, wash buffer, chrongen solution A, Chromogen solution B, Stop solution, Instruction for user (IFU) ; b.*Freshly distilled or deionized water, disposable gloves and timer, appropriate waste containers for potentially contaminated materials, dispensing system and/or pipette, disposable pipette tips, absorbent tissue or clean towel, dry incubator or water bath, 37 ± 1 °C, plate reader, single wavelength 450 nm or dual wavelength 450/600 ~ 650 nm, microwell aspiration/wash system;. c*:Sampling tube, centrifuge for plasmas, micropipettes, stopwatch, lancets for capillary samples; d:IFU, cassettes, buffer, capillary tubes.

### Reference test: WANTAI SARS-CoV-2 Ab ELISA

WANTAI SARS-CoV-2 Ab ELISA is an Enzyme-Linked Immunosorbent Assay (ELISA) intended for qualitative detection of total antibodies (including IgG and IgM) to SARS-CoV-2 in human serum or plasma [24]. It is a two-step incubation antigen “sandwich” enzyme immunoassay kit, which uses polystyrene microwell strips pre-coated with recombinant SARS-CoV-2 antigen. The antigen used in the assay is the receptor-binding domain of SARS-CoV-2 spike protein. Patient’s serum or plasma specimen is added, and during the first incubation, the specific SARS-CoV-2 antibodies will be captured inside the wells if present [24]. The microwells are then washed to remove unbound serum proteins. Second recombinant SARS-CoV-2 antigen conjugated to the enzyme Horseradish Peroxidase (HRP-Conjugate) is added, and during the second incubation, the conjugated antigen will bind to the captured antibody inside the wells. The microwells are then washed to remove unbound conjugate, and Chromogen solutions are added into the wells. In wells containing the antigen-antibody-antigen (HRP) “sandwich” immunocomplex, the colorless Chromogens are hydrolyzed by the bound HRP conjugate to a blue colored product. The blue color turns yellow after the reaction is stopped with sulfuric acid. The amount of color intensity can be measured and it is proportional to the amount of antibody captured inside the wells, and to the specimen respectively. Wells containing specimens negative for SARS-CoV-2 antibodies remain colorless. According to the manufacturer (Beijing Wantai Biological), clinical validation study of WANTAI SARS-CoV-2 Ab ELISA was observed that the detection rate of the test was closely related to the time of disease onset, the test showed higher positive detection rate in specimens from patients with long time post onset of first symptom. The test sensitivity was 55,38% for less than 7 days from symptoms; 84,78% between 8 and 14 day from symptoms and 98,72% for more than 15 days from symptoms [24]. In addition to the performance provided by WANTAI SARS-CoV-2 Ab ELISA compared to others [9, 10].

### Panel plasma analysis

The serological RDTs were evaluated using the WANTAI SARS-CoV-2 Ab ELISA positive and negative specimen (plasma). All tests were used according to the manufacturers’ specifications and the Good Laboratory Practices (GLP). Due to insufficient numbers of tests, the STANDARD Q COVID-19 IgM/IgG Combo and Panbio COVID-19 IgG/IgM RAPID test device were evaluated with only 60 positive and 60 negative samples, compared to 157 positive and 157 negative samples for the other eight RDTs. To avoid comparison of results between tests during laboratory analysis, each rapid test under evaluation was tested in one run with all samples in the panel before moving on to another test. The RDT result was considered positive if it detected IgG and/or IgM antibodies, and negative if no antibodies were detected.

### Statistical analysis

Data were entered into Excel and then analyzed using OpenEpi software The results obtained with the serological RDT were compared with those of the ELISA, and the main performance characteristics of the RDT were determined. For this purpose, the results of each RDT were classified into 2 categories (positive or negative results). In relation to the known results of the serological ELISA (reference to the serological RDT), the RDT results were classified into true positives (TP), false positives (FP), true negatives (TN), and false negatives (FN) on a double entry contingency table (Table 2). Test sensitivity (capacity to capture all true positives) was calculated according to the formula (TP)/(TP + FN), and diagnostic specificity (capacity to rull out all true negatives) was calculated according to the formula (TN)/(TN + FP). In addition to the two main characteristics (Sensitivity and Specificity) of the diagnostic performance of the test, other test-specific parameters such as positive predictive value (PPV, the probability that the plasma sample has the COVID-19 antibodies when restricted to those plasma who test positive) and negative predictive value (NPV, the probability that the plasma sample has not the COVID-19 antibodies when restricted to those plasma who test negative): PPV = TP/TP + FP and NPV = TN/TN + FN); the positive and negative likelihood ratios (LRP and LRN); and the Kappa Coefficient of agreement between RDT and ELISA. These characteristics were calculated with their 95% confidence intervals. The results of these calculations were expressed as a percentage. The Kappa coefficient of agreement was interpreted according to the criteria of Landis and Koch [25] as follows: Kappa < 0, no agreement; 0 < kappa ≤ 0.2, slight agreement; 0.2 < kappa < 0.4, fair agreement; 0.4 < kappa ≤ 0.6,moderate agreement; 0.6 < kappa ≤ 0.8, substantial agreement; 0.8 < kappa ≤ 1, near perfect agreement.

**Table 2:** Cross tabulation of the index test results by the reference standard’s results

Index tests	Results	WANTAI SARS-CoV-2 Ab ELISA		
		Positive (n)	Negative (n)	Total
COVID-19 IgG/IgM Rapid Test (Whole blood/Serum/Plasma) Sienna TM	Positive	*71*	*03*	*74*
	Negative	*86*	*154*	*240*
Total		157	157	314
COVID-19 BSS (IgG/IgM), Biosynex	Positive	76	01	77
	Negative	81	156	237
Total		157	157	314
COVID-19 IgG/IgM cassette (whole blood/serum/plasma) ACCU-Tell	Positive	64	01	65
	Negative	93	156	249
Total		157	157	314
COVID-19 IgG/IgM Rapid Test (whole blood/serum/plasma) InnoScreen™	Positive	77	04	81
	Negative	80	153	233
Total		157	157	314
COVID-19 IgG/IgM Rapid test device (WB/S/P) Safecare Bio-Tech	Positive	62	03	65
	Negative	95	154	249
Total		157	157	314
COVID-19 IgG/IgM Rapid Test (Whole blood/Serum/Plasma) Manufacturer name was missing (anonymous)	Positive	77	01	78
	Negative	80	156	236
Total		157	157	314
2019-nCOV IgG/IgM Rapid test Device Hangzhou Real Tech	Positive	43	03	46
	Negative	114	154	268
Total		157	157	314
*COVID-19 IgG/IgM Rapid Test Cassette (Whole blood/Serum/Plasma)* *Zhejiang Orient Gene Biotech Co.,Ltd*	Positive	81	04	85
	Negative	76	153	229
Total		157	157	314
STANDARD Q COVID-19 IgM/IgG Combo^*^ SD Biosensor	Positive	37	00	37
	Negative	23	60	83
Total		60	60	120
Panbio COVID-19 IgG/IgM RAPID test device^*^ Abbott	Positive	24	04	28
	Negative	36	56	92
Total		60	60	120

^*^ STANDARD Q COVID-19 IgM/IgG Combo (SD BIOSENSOR) and Panbio COVID-19 IgG/IgM RAPID test device (Abbott) were evaluated with 120 samples of which 60 were positive and 60 were negative for WANTAI SARS-CoV-2 Ab ELISA.

## Results

### Test performances

Tables 2 and 3 show the comparison results between the rapid tests and the reference test. Compared to the WANTAI SARS-CoV-2 Ab ELISA test, the results generally show that the serological RDTs have specificities ranging from 93.33 to 100%. However, all the RDTs evaluated had a sensitivity of less than 65%. The lowest sensitivity was 27.39% (21.02–34.84) observed with the 2019-nCOV IgG/IgM Rapid test Device (HANGZHOU REALY TECH), and the highest was 61.67% (49.02–72.91) obtained for the STANDARD Q COVID-19 IgM/IgG Combo SD Biosensor. For nine of the ten RDTs, the sensitivity was less than 50% compared to the reference test. These are COVID-19 IgG/IgM Rapid Test, Sienna TM (T1); COVID-19 BSS (IgG/IgM) Biosynex (T2); COVID-19 IgG/IgM cassette (plasma) ACCU-Tell (T3); COVID-19 IgG/IgM Rapid Test, InnoScreen™ (T4); COVID-19 IgG/IgM Rapid test device, Safecare Bio-Tech (T5); COVID-19 IgG/IgM Rapid Test (T6); 2019-nCOV IgG/IgM Rapid test Device Hangzhou Realy Tech (T7), COVID-19 IgG/IgM Rapid Test Cassette, Zhejiang Orient Gene Biotech Co.,Ltd (T8) STANDARD Q COVID-19 IgM/IgG Combo SD Biosensor (T9); Panbio COVID-19 IgG/IgM RAPID test device, Abbott (T10).

The negative predictive value ranged from 57.46% (51.48–63.24) for the least sensitive test to 72.29% (61.84–80.77) for the most sensitive. As for the positive predictive values (PPV), the lowest was 85.71% (68.51–94.3) for the least specific test (Panbio™ COVID-19 IgG/IgM Rapid test device) to 100% for the most specific (STANDARD Q COVID-19 IgM/IgG Combo SD Biosensor). Two tests had a kappa value of agreement with ELISA test between 0.2 and 0.4 (COVID-19 IgG/IgM RAPID TEST DEVICE (WB/S/P) Safecare Bio-Tech and 2019-nCOV IgG/IgM Rapid test Device Hangzhou Realy Tech). While the concordance of eight tests with WANTAI SARS-CoV-2 Ab ELISA test was between 0.41 and 0.6. The only test that recorded a Kappa coefficient value greater than 0.6 was the STANDARD Q COVID-19 IgM/IgG Combo SD Biosensor. (Table 3).

**Table 3:** Estimates of diagnostic accuracy and their precision (such as 95% confidence intervals)

	COVID-19 IgG/IgM Rapid Test Sienna TM		COVID-19IgG/IgM Rapid Test Biosynex		COVID-19 IgG/IgM Cassette ACCU-Tell		COVID-19 IgG/IgM Rapid Test InnoScreen™		COVID-19 IgG/IgM Rapid test Device WB/S/P Safecare Bio-Tech	
Parameters	Estimate	95%CI	Estimate	95%CI	Estimate	Estimate	Estimate	95%CI	Estimate	95%CI
Sensitivity (%)	45.22	37.64–53.03	48.41	40.72–56.17	39.49	39.49	49.04	41.34–56.79	39.49	32.18–47.3
Specificity (%)	98.09	94.53–99.35	99.36	96.48–99.89	98.09	98.09	97.45	93.63-99.0	98.09	94.53–99.35
*PPV (%)*	95.95	88.75–98.61	98.7	93,0-99.77	95.38	95.38	95.06	87.98–98.06	95.38	87.29–98.42
*NPV (%)*	64.17	57.92–69.97	65.82	59.57–71.57	61.85	61.85	65.67	59.36–71.46	61.85	55.68–67.66
*PLR*	23.67	11.91–47.03	76.0	10.42–55.46	20.67	20.67	19.25	11.49–32.26	20.67	10.24–41.69
*NLR*	0.56	0.54–0.57	0.52	0.51–0.53	0.62	0.62	0.52	0.51–0.53	0.62	0.60–0.63
Kappa	0.4331	0.34–0.52	0.48	0.38–0.57	0.37	0.37	0.46	0.37–0.56	0.37	0.29–0.46
Accuracy (%)	71.66	66.43–76.36	73.89	68.76–78.43	68.79	68.79	73.25	68.09–77.84	68.79	63.46–73.66
	**COVID-19 IgG/IgM Rapid** **Test Whole** **blood/Serum/Plasma**		**2019-nCOV IgG/IgM Rapid** **test Device** **Hangzhou Realy Tech**		**COVID-19 IgG/IgM Rapid** **Test (Zhejiang Orient Gene** **Biotech Co.-Ltd)**		**STANDARD Q COVID-19** **IgM/IgG Combo** **SD Biosensor**		**Panbio COVID-19 IgG/IgM** **Rapid test Device** **Abbott**	
Parameters	Estimate	95%CI	Estimate	95%CI	Estimate	95%CI	Estimate	95%CI	Estimate	95%CI
Sensitivity (%)	49.04	41.34–56.79	27.39	21.02–34.84	51.59	43.83–59.28	61.67	49.02–72.91	40	28.57–52.63
Specificity (%)	99.36	96.48–99.89	98.09	94.53–99.35	97.45	93.63-99	100	93.98–100	93.33	84.07–97.38
*PPV (%)*	98.72	93.09–99.77	93.48	82.5- 97.76	95.29	88.52–98.16	100	90.59–100	85.71	68.51–94.3
*NPV (%)*	66.1	59.85–71.84	57.46	51.48–63.24	66.81	60.48–72.59	72.29	61.84–80.77	60.87	50.65–70.21
*PLR*	77.0	10.56–561.3	14.33	6.61–31.09	20.25	12.13–33.81	Undefined	undefined’.	6.0	3.25–11.07
*NLR*	0.51	0.50–0.52	0.74	0.73–0.75	0.50	0.48–0.51	0.38	0.35–0.41	0.64	0.61–0.68
Kappa	0.48	0.39–0.58	0.25	0.18–0.33	0.49	0.39–0.59	0.62	0.45–0.78	0.33	0.18–0.48
Accuracy (%)	74.2	69.09–78.73	62.74	57.27–67.9	74.52	69.43–79.03	80.83	72.88–86.88	66.67	57.83–74.47

Legend: Se: Sensitivity, Sp: Specificity, PPV: positive predictive value, NPV = negative predictive value, PLR: positive likelihood ratio, NLR: negative likelihood ratio.

## Discussion

This study evaluated the performance of rapid serological tests (RDTs) for SARS-CoV-2 compared to the WANTAI SARS-CoV-2 Ab ELISA test as reference. It shows that the performance of serological RDTs ranged from 27.39 to 61.67% for sensitivity, while specificity varied from 97.45 to 100% depending on the brand of the test. The highest sensitivities in our study were obtained for COVID-19 IgG/IgM Rapid Test, Zhejiang Orient Gene Biotech Co., Ltd and STANDARD Q COVID-19 IgM/IgG Combo SD BIOSENSOR with 51.59% and 61.67% respectively. These two tests also ranked with the highest specificities. Of note, none of the tests evaluated had reached the sensitivity announced by the manufacturer.

The literature reported that most rapid serological tests have lower sensitivity than ELISA tests [22, 26, 27]. The sensitivities of COVID-19 ELISA tests for IgG/IgM or IgG and IgM ranged from 75 to 93% depending to the studies, while for rapid tests they ranged from 36 to 100%. The specificities reported in these studies were similar between ELISA serological tests (91.9–100%) and rapid tests (89% and 100%) [27]. In a systematic review, Lisboa Bastos et al.,. reported lower combined sensitivities for serological RDTs (66%, 95%CI: 49.3–79.3) than for ELISAs (84.3%, 95%CI: 75.6–90.9) [26]. In general, the weak sensitivity of serological tests are more marked in asymptomatic subjects than in symptomatic subjects because the production of SARS-CoV-2 antibodies would be greater in symptomatic subjects than in asymptomatic ones [28]. Mercado et al. evaluated the clinical performance of nine serological RDTs compared to RT-PCR and found that their sensitivity was less than 40% in asymptomatic patients [8]. In symptomatic subjects, however, the sensitivity of the tests ranged from 0,0 to 64.2% for IgM and 11.11-33.30% for IgG during the first 8 days of symptoms, and from 37.50 to 93.75% for IgM and 70.83-93.75% for IgG between 8^ème^ and 11^ème^ days [8]. Another study evaluating serological tests including RDTs also found that these had sensitivities ranging from 51.80 to 67.90%, and specificities ranging from 95.6 to 100.0%. [29]. Vásárhelyi B, Kristóf et al. obtained even lower sensitivities of 33.30% and 35.48% for the Ahui and Clungene tests respectively [7], comparable to the sensitivity of some of the RDTs evaluated in our study.

In addition to the notion of symptoms, the performance of COVID-19 serological RDTs compared to ELISA tests may vary between brands/manufacturers. A study comparing the performance of COVID-19 serological RDTs and ELISA tests in the detection of SARS-CoV-2 antibodies in subjects who have been symptomatic for more than 14 days found high sensitivity for some RDTs (> 95% for some RDTs (ACRO Biotech and VivaChek Laboratories), comparable to that of ELISA methods (96% for WANTAI SARS-CoV-2 Ab ELISA and Vircell® IgG), while other RDTs showed lower sensitivity (66.7% for Coris-Bioconcept) [9]. However, this study involved a very limited number of samples.

Regarding RDTs specificities, except for the Panbio™ COVID-19 IgG/IgM (Abbott) (Sp: 93.33%), the evaluated tests, showed good specificity in the detection of SARS-CoV-2 antibodies (specificity ≥ 95% compared to WANTAI SARS-CoV-2 Ab ELISA). The specificities reported in our study show that the RDTs evaluated have a high probability of detecting negative subjects, and providing few false-positive results. Several studies had already concluded that the specificities of the serological RDTs varied widely. Some studies have reported specificities close to 98% (96.7% for WONDFO®) while others have reported specificities close to 50% [11] while others report lower specificities (72.85% for Ahui and 85.02% for Clungene) [7].

The high specificity of the RDTs evaluated in our study reinforce their positive predictive values (PPV). These positive predictive values, defined as the probability that the subject tested positive using the test is indeed positive for SARS-CoV-2 antibodies, ranged from 85.71 to 100%. The negative predictive value (NPV) ranged from 66.1 to 72.29%, representing the probability that subjects who tested negative with the index tests were negative for SARS-CoV-2 antibodies. Vásárhelyi B, Kristóf et al., in 2020 found PPVs of 7.28% and 13.13% for Ahui and Clungene respectively, [7] ; even lower values than our study.

Agreement between serological RDTs and the WANTAI SARS-CoV2 Ab ELISA was ‘fair’ for nine of the ten tests (kappa = 0.25 to 0.49), and ‘moderate’ for only one test, the STANDARD Q COVID-19 IgM/IgG Combo SD Biosensor (kappa = 0.61). The latter has the best overall value in the detection of SARS-CoV-2 antibodies with an estimated diagnostic accuracy of 80.83%.

Our study has a number of limitations. The reference test used was the WANTAI SARS-CoV2 Ab ELISA for the qualitative detection of total antibodies to SARS-CoV-2 in human specimens. It does not allow separate assessment of the sensitivity and specificity of IgG and IgM of each of the ten index tests. Also, it is recognized that the detection rate of serological tests is closely related to the presence or absence of symptoms and the time of onset of symptoms, which our study did not report. Finally, most of the studies found in the literature on the evaluation of serological tests have used samples taken from symptomatic patients after a RT-PCR positivity of at least 7 to 21 days as reference. Despite these limitations, our study, which directly uses an ELISA test as a reference for rapid serological tests, is providing information to guide the choice and use between various types of serological tests in different contexts, such as in seroprevalence studies often performed in populations independently of the notions of history or delay of symptoms of COVID-19.

## Conclusion

The COVID-19 serological RDTs evaluated in this study show variable, and low, sensitivities compared to the WANTAI COVID-19 Ab ELISA as reference. No tests meet the 95% sensitivity criteria required for use in the serological diagnosis of COVID-19, regardless of history or time of onset of COVID-19 symptoms. On the other hand, the specificity of RDTs compared to WANTAI COVID-19 Ab ELISA remains relatively good. The results of this study should be interpreted with caution because serological tests generally have a better positive detection rate in specimens from symptomatic patients with a long period after the onset of symptoms. However, ours finding may have implications for the interpretation and comparison of COVID-19 seroprevalence studies depending on the type of test used.

## Data Availability

The data sets used and analyzed during the current study are available from the corresponding author on reasonable request.

## Abbreviations

Ab: Antibody
COVID-19: Coronavirus disease 2019
ELISA: Enzym Linked ImmunoSorbent Assay
FN: False negative
FP: False positive
LRN: Likelihood Ratio of Negative Test
LRP: Likelihood Ratio of Positive Test;
NPV: Negative Predictive Value;
PPV: Positive Predictive Value;
RDT: Rapid diagnostic test
RT-PCR: Reverse transcriptase polymerase chain reaction
SARS-CoV-2: Severe acute respiratory syndrome coronavirus 2
TN: True negative
TP: True positive
